# Association of COVID-19 Vaccination During Pregnancy With Incidence of SARS-CoV-2 Infection in Infants

**DOI:** 10.1001/jamainternmed.2022.2442

**Published:** 2022-06-01

**Authors:** Ellen Øen Carlsen, Maria C. Magnus, Laura Oakley, Deshayne B. Fell, Margrethe Greve-Isdahl, Jonas Minet Kinge, Siri E. Håberg

**Affiliations:** 1 Centre for Fertility and Health, Norwegian Institute of Public Health, Oslo, Norway; 2 Department of Non-communicable Disease Epidemiology, London School of Hygiene and Tropical Medicine, London, England; 3 School of Epidemiology and Public Health, University of Ottawa, Ottawa, Ontario, Canada; 4 Children’s Hospital of Eastern Ontario Research Institute, Ottawa, Ontario, Canada; 5 Department of Infectious Disease, Norwegian Institute of Public Health, Oslo, Norway; 6 Department of Health Management and Health Economics, Institute of Health and Society, University of Oslo, Oslo, Norway

## Abstract

**Importance:**

Pregnant women are recommended to receive COVID-19 vaccination to reduce risk of severe COVID-19. Whether vaccination during pregnancy also provides passive protection to infants after birth remains unclear.

**Objective:**

To determine whether COVID-19 vaccination in pregnancy was associated with reduced risk of COVID-19 in infants up to age 4 months during COVID-19 pandemic periods dominated by Delta and Omicron variants.

**Design, Setting, and Participants:**

This nationwide, register-based cohort study included all live-born infants born in Norway between September 1, 2021, and February 28, 2022.

**Exposures:**

Maternal messenger RNA COVID-19 vaccination during second or third trimester compared with no vaccination before or during pregnancy.

**Main Outcomes and Measures:**

The risk of a positive polymerase chain reaction test result for SARS-CoV-2 during an infant’s first 4 months of life by maternal vaccination status during pregnancy with either dose 2 or 3 was estimated, as stratified by periods dominated by the Delta variant (between September 1 and December 31, 2021) or Omicron variant (after January 1, 2022, to the end of follow-up on April 4, 2022). A Cox proportional hazard regression was used, adjusting for maternal age, parity, education, maternal country of birth, and county of residence.

**Results:**

Of 21 643 live-born infants, 9739 (45.0%) were born to women who received a second or third dose of a COVID-19 vaccine during pregnancy. The first 4 months of life incidence rate of a positive test for SARS-CoV-2 was 5.8 per 10 000 follow-up days. Infants of mothers vaccinated during pregnancy had a lower risk of a positive test compared with infants of unvaccinated mothers and lower risk during the Delta variant–dominated period (incidence rate, 1.2 vs 3.0 per 10 000 follow-up days; adjusted hazard ratio, 0.29; 95% CI, 0.19-0.46) compared with the Omicron period (incidence rate, 7.0 vs 10.9 per 10 000 follow-up days; adjusted hazard ratio, 0.67; 95% CI, 0.57-0.79).

**Conclusions and Relevance:**

The results of this Norwegian population-based cohort study suggested a lower risk of a positive test for SARS-CoV-2 during the first 4 months of life among infants born to mothers who were vaccinated during pregnancy. Maternal COVID-19 vaccination may provide passive protection to young infants, for whom COVID-19 vaccines are currently not available.

## Key Points

**Question:**

Is maternal COVID-19 vaccination during the second or third trimester of pregnancy associated with reduced risk of COVID-19 within the first 4 months of life in their infants?

**Findings:**

In this register-based cohort study of all live-born infants in Norway, there was a lower incidence of a positive SARS-CoV-2 test result in infants born to women vaccinated with a messenger RNA vaccine during pregnancy. The risk was lower during the period dominated by the Delta variant than during the Omicron-dominated period.

**Meaning:**

The study results suggest that maternal COVID-19 vaccination during pregnancy could protect against infant SARS-CoV-2 infection in the early months of life.

## Teaser

This cohort study examines the association of COVID-19 vaccination during pregnancy with reduced risk of COVID-19 in Norwegian infants up to age 4 months during COVID-19 pandemic periods dominated by Delta and Omicron variants.

## Introduction

The risk of critical illness because of COVID-19 has been reported to be higher in infants younger than 1 year compared with older children.^1^ To our knowledge, no COVID-19 vaccines are currently available for infants or children younger than 5 years. Transplacental transfer of maternal vaccine-derived antibodies against pertussis and seasonal influenza has been demonstrated following vaccination during pregnancy, and maternal immunization provides passive protection against infection to infants during the first months after birth.^2,3^ It is plausible that COVID-19 vaccination during pregnancy could provide passive protection from COVID-19 to infants during their first months of life.^4^ Vaccine-derived maternal antibodies have been identified in cord blood after COVID-19 vaccination during pregnancy, and a recent study found maternal vaccination to be associated with a 61% reduced risk of infant hospitalization for COVID-19.^5,6,7^ This study evaluated the association between maternal COVID-19 vaccination during pregnancy and incidence of infant SARS-CoV-2 infection during the first 4 months of life, as well as whether the association differed according to Delta variant and Omicron variant–dominated time periods.^8,9,10,11^

## Methods

### Ethical Approval

This study followed the Strengthening the Reporting of Observational Studies in Epidemiology (STROBE) reporting guidelines and was approved by the Regional Committee for Medical and Health Ethics of South/East Norway. This committee waived the need for informed consent from participants in this registry-based study.

### Study Population and Data Sources

**Figure 1.**
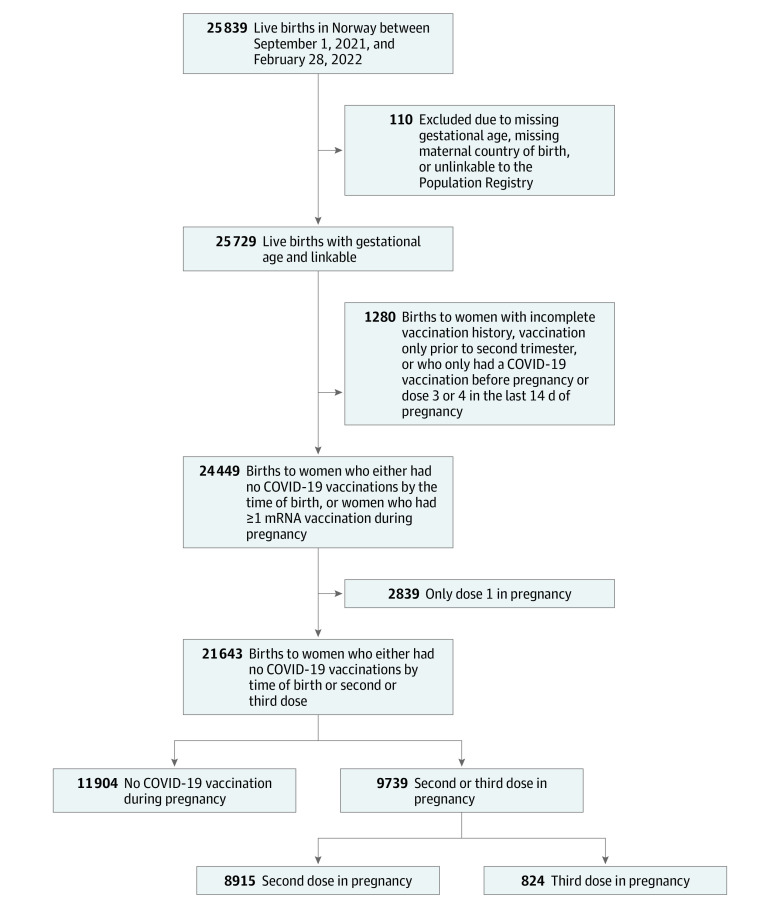
Flowchart of Selection of Study Participants

All live births in Norway between September 1, 2021, and February 28, 2022, were identified in the Medical Birth Registry of Norway (Figure 1),^12^ which captures all pregnancies ending after completion of gestational week 12. Newborns were excluded if the mother or infant did not have a permanent national identification number, which was used to link information across registries.

All data used in this study were provided by the Emergency Preparedness Register for COVID-19 (Beredt C19),^13^ which is run by the Norwegian Institute of Public Health. This register was established in response to the COVID-19 pandemic in 2020 in accordance with the Health Preparedness Act §2-4 and contains daily updated data from the Norwegian health registries.

### COVID-19 Vaccination During Pregnancy

The Norwegian Immunization Register^14^ contains registrations of all COVID-19 vaccinations, including dates of all doses and the type of vaccine product. Vaccine doses reported fewer than 20 days after the previous dose were not included. Women who received a second or third dose of a messenger RNA (mRNA) vaccine after gestational day 83 and up to 14 days before delivery were considered vaccinated. Infants born to women who received their third or fourth vaccine dose between 13 and 7 days before birth were excluded, as they would be censored before birth in the statistical analyses. We excluded infants born to women vaccinated outside Norway while pregnant and those vaccinated exclusively before pregnancy or who only received dose 1 during pregnancy because the maternal antibody level and possible transplacental transfer of antibodies for these scenarios are uncertain.^15,16,17^

### SARS-CoV-2 Infection

During the study period (September 1, 2021, to April 4, 2022), the date of testing and results of all SARS-CoV-2 polymerase chain reaction (PCR) tests were registered in the Norwegian Surveillance System for Communicable Diseases.^18^ We included the first positive PCR test for SARS-CoV-2 registered at least 1 day after birth and within 122 days after birth (4 months of age). Similarly, we identified women with a positive SARS-CoV-2 test 14 days or more before giving birth. In Norway, PCR tests were free of charge and widely available.

### Covariates

From the Medical Birth Registry of Norway, we derived maternal age at conception (<24, 25-29, 30-34, 35-39, ≥40 years) and extracted information on parity (0, 1, or ≥2) and calendar week of birth. The registered gestational age in the birth registry was estimated from routine ultrasonography assessments or last menstrual period if there were no ultrasonography estimates. From the Population Registry of Norway, we obtained information on maternal country of birth (Scandinavian country [Norway, Denmark, and Sweden], or non-Scandinavian countries) and current county of residence (Oslo, Viken, or other Norwegian county). From Statistics Norway we obtained information on the highest maternal educational level as of 2019 (no higher education, higher education ≤4 years, >4 years of higher education, or missing).

### Statistical Analysis

We calculated incidence rates of SARS-CoV-2 infection (number of infants with positive tests among all infants at risk at the day of testing) in infants within 4 months after birth by maternal vaccination status. We split the follow-up time on December 31, 2021, by introducing a variable with 2 categories that corresponded to the time before this date (proxy for the Delta-dominated period) and after this date (proxy for the Omicron-dominated period). Using a Cox proportional hazards model, we estimated hazard ratios (HRs) for infant SARS-CoV-2 infection using calendar time (in days) as the time axis. Infant follow-up began on the date of birth. Observations were censored at age 4 months, death or emigration, date of when a woman received a vaccine dose outside Norway, or on April 4, 2022, whichever came first. For women vaccinated at the end of pregnancy or after pregnancy, infants were censored 14 days after the vaccination date of dose 1 and 7 days after dose 3 or 4. This was done because maternal vaccination within the last 14 days of pregnancy could potentially provide an exclusive protective effect from antibody transfer through breastfeeding or cocooning (ie, immunizing primary caretakers).^19,20^

We assessed differences in the Delta and Omicron periods by using linear combinations of the coefficients for vaccination status and an interaction term between vaccination status and period. Multivariable analyses adjusted for maternal age at conception, parity, educational level, county of residence, and maternal country of birth.

### Sensitivity Analyses

First, we excluded infants born to women who had a positive SARS-CoV-2 test more than 14 days before delivery, as women with a history of COVID-19 might be less likely to become vaccinated during pregnancy and could transfer anti–SARS-CoV-2 antibodies across the placenta.^5^ Second, in separate analyses, we restricted the sample to (1) term-born infants, (2) infants who had the opportunity to reach 42 completed gestational weeks by the end of the inclusion period to avoid oversampling preterm births, (3) infants born to Scandinavian-born women, (4) infants born to first-time mothers, and (5) infants born to women who only received mRNA vaccines for all doses. Third, to obtain more robust results for the Omicron-dominated period, we used an alternative follow-up period starting on January 15, 2022, as after this date the circulating virus was more certain to be Omicron. Finally, as testing and risk of infection may differ by infant age, we used infant age in days as the time axis, while adjusting for week of birth.

### Secondary Analyses

Using the Norwegian Patient Registry,^21^ we explored the risk of infant hospitalization for COVID-19. All hospital admissions were registered with admission and discharge dates, as well as diagnostic codes, using the *International Statistical Classification of Diseases and Related Health Problems, Tenth Revision (ICD-10)*. We identified all infants with a hospitalization with the *ICD-10* code U07.1 (COVID-19, virus identified) as the main diagnosis within the first 4 months of life and reported the crude proportions by maternal vaccination status.

To assess associations between maternal vaccination, even if not fully vaccinated, and incidence of a positive PCR test for SARS-CoV-2 among infants, we conducted a secondary analysis in which we compared the risk of a positive SARS-CoV-2 test in infants born to unvaccinated women with those with mothers who had received a first dose of an mRNA vaccine during the second or third trimester (these were excluded from the main analysis). Infants were censored 14 days after a woman received the second dose postpartum or during the last 14 days of pregnancy and 14 days after the first dose among the unvaccinated group. We compared the timing of vaccination in pregnancy in those who received only 1 dose in pregnancy with those who were fully vaccinated. Furthermore, we conducted a secondary analysis stratifying on whether the woman received COVID-19 vaccine dose number 2 or 3 during pregnancy.

To explore possible differences in the likelihood of being tested in infants among vaccinated and unvaccinated mothers, we assessed the proportions in each group that had at least 1 registered SARS-CoV-2 PCR test (positive or negative) before age 4 months. We calculated crude incidence rates for registration of a PCR test by maternal vaccination status. Again, infants were censored at time of death, emigration, or maternal receipt of a vaccine dose outside Norway. All analyses were conducted using Stata, version 16.0 SE (StataCorp).

## Results

Of 21 643 newborns included in the study, 9739 (45%) were born to women who received a second or third dose of a COVID-19 mRNA vaccine during the last 2 trimesters of pregnancy (Table 1). Fewer than 5 women received a fourth dose in pregnancy. Compared with vaccinated mothers, unvaccinated mothers were younger, had higher parity and lower education, and fewer were born in Scandinavia. The proportion of infants born to a vaccinated mother increased during the study period (eFigure 1A in the Supplement). Most newborns with a positive SARS-CoV-2 test during the fall of 2021 were born to unvaccinated mothers (eFigure 1B in the Supplement), but an infant’s age at the time of a positive test was similar between the groups (eFigure 1C in the Supplement). The number of tested infants decreased by mid-February 2022 (eFigure 1D in the Supplement).

**Table 1.** Characteristics of the Study Population

Characteristic	No. (%)	
	Unvaccinated	Vaccinated with second or third dose during the last 2 trimesters of pregnancy
Live-born infants^a^	11 904 (55.0)	9739 (45.0)
Maternal age at conception, median (IQR), y	30.2 (26.9-33.6)	31.2 (28.4-34.3)
<24	1628 (13.7)	697 (7.2)
25-29	4155 (34.9)	3036 (31.2)
30-34	4064 (34.1)	4036 (41.4)
35-39	1729 (14.5)	1724 (17.7)
≥40	328 (2.8)	246 (2.5)
Maternal parity		
0	4919 (41.3)	4430 (45.5)
1	4264 (35.8)	3643 (37.4)
≥2	2721 (22.9)	1666 (17.1)
Maternal country of birth		
Scandinavia	7267 (61.1)	8434 (86.6)
Outside Scandinavia	4637 (39.0)	1305 (13.4)
Maternal education		
No higher education	4858 (40.8)	2477 (25.4)
≤4 y Of higher education	3577 (30.1)	4216 (43.3)
>4 y Of higher education	1433 (12.0)	2540 (26.1)
Missing	2036 (17.1)	506 (5.2)
Preterm births	621 (5.2)	599 (6.2)
Vaccine dose <14 d before birth or after birth^b^	7112 (59.7)	3026 (31.1)
Positive SARS-CoV-2 test in infant^c^	496 (4.2)	410 (4.2)
Positive test in infant		
Before January 1, 2022	146 (1.5)	25 (0.5)
From January 1, 2022	350 (5.2)	385 (4.0)
Age at first positive SARS-CoV-2 test in infant, mean (SD), d	59.8 (31.6)	62.2 (31.9)
Hospitalization with COVID-19 as main diagnosis before age 4 mo^c^	8 (0.1)	7 (0.1)
Maternal COVID-19 before end of pregnancy	1036 (8.7)	363 (3.7)
Part of a multiple birth	282 (2.4)	296 (3.0)
At least 1 infant SARS-CoV-2 PCR test performed before age 4 mo	2309 (19.4)	1206 (12.4)

Abbreviation: PCR, polymerase chain reaction.

^a^ A total of 21 643 live infants born in Norway between September 1, 2021, and February 28, 2022.

^b^ Received within 4 months postpartum.

^c^ During the entire follow-up period.

### Incidence of SARS-CoV-2 in Infants

**Figure 2.**
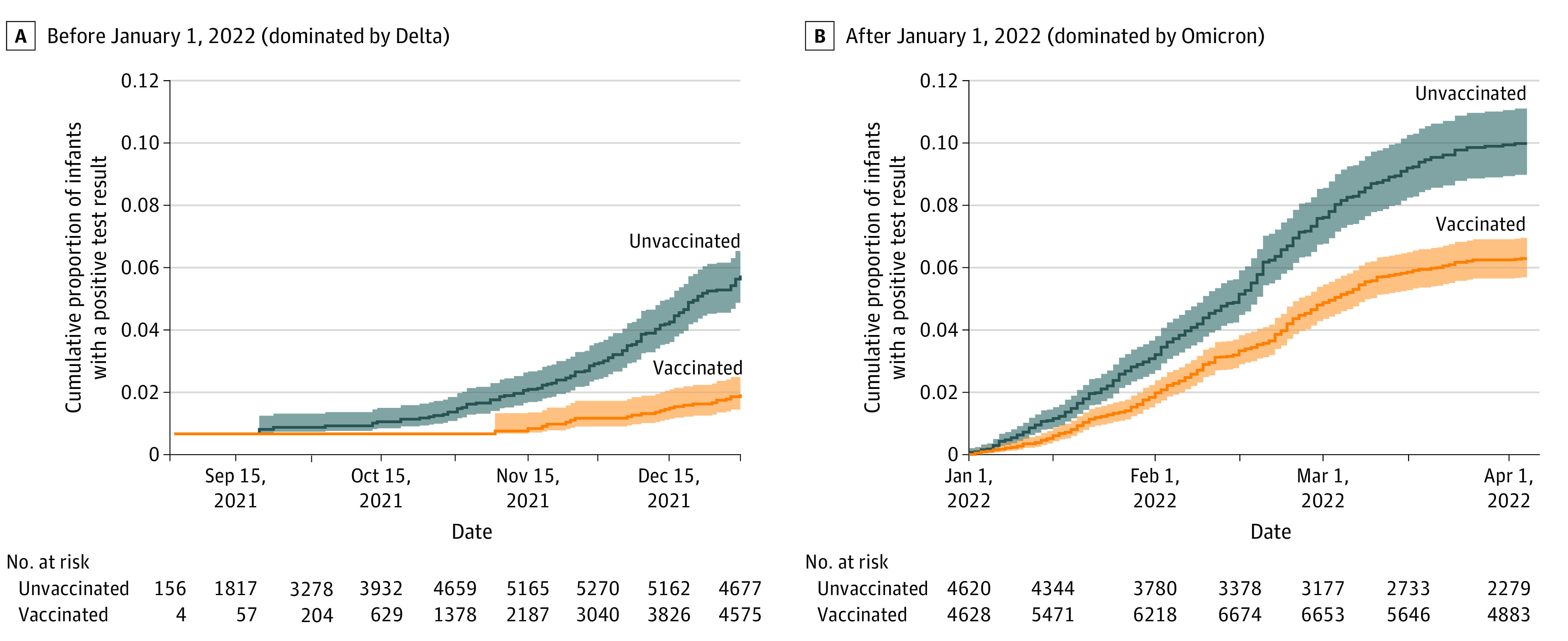
Cumulative Proportion of Number of Infants With a Positive SARS-CoV-2 Polymerase Chain Reaction Test by Maternal COVID-19 Vaccination Status A, Delta variant–dominated period: September 1 to December 31, 2021. B, Omicron variant–dominated period: January 1 to April 4, 2022. The shaded bands correspond to the 95% CIs.

A total of 906 infants (4.1%) were registered with a positive PCR test for SARS-CoV-2 during the first 4 months of life. Infants born to vaccinated mothers had a lower incidence of SARS-CoV-2 (Figure 2). During the Delta-dominated period (before January 1, 2022), crude incidence rates for a positive test were 1.2 per 10 000 follow-up days among infants born to vaccinated mothers and 3.0 per 10 000 follow-up days among infants born to unvaccinated mothers. The corresponding adjusted HR (aHR) for the Delta-dominated period was 0.29 (95% CI, 0.19-0.44) (Table 2). During the Omicron-dominated period (starting on January 1, 2022), the crude incidence rates for a positive SARS-CoV-2 test were 7.0 per 10 000 follow-up days among infants born to vaccinated mothers and 10.9 per 10 000 follow-up days among infants born to unvaccinated mothers (aHR, 0.67; 95% CI, 0.57-0.79) (Table 2). We observed no violation of the proportional hazard assumption.

**Table 2.** Hazard Ratios of a Positive SARS-CoV-2 PCR Test in Infants Before Age 4 Months

Characteristic	Before January 1, 2022						After January 1, 2022					
	Live-born infants, No.	Follow-up time, d	COVID-19 positive test, No. (%)	Incidence rate, cases/10 000 d of follow-up	Hazard ratio (95% CI)^a^		Live-born infants, No.	Follow-up time, d	COVID-19 positive test, No. (%)	Incidence rate, cases/10 000 d of follow-up	Hazard ratio (95% CI)^a^	
					Unadjusted	Adjusted^b^					Unadjusted	Adjusted^b^
Unvaccinated	9759	485967	146 (1.5)	3.0	1 [Reference]	1 [Reference]	6728	320292	350 (5.2)	10.9	1 [Reference]	1 [Reference]
Vaccinated	4696	206857	25 (0.5)	1.2	0.29 (0.19-0.44)	0.29 (0.19-0.46)	9616	546790	385 (4.0)	7.0	0.63 (0.55-0.73)	0.67 (0.57-0.79)

Abbreviation: PCR, polymerase chain reaction.

^a^ Infants born to women who completed their 2-dose or 3-dose vaccination series during the second or third trimesters of pregnancies were compared with infants born to unvaccinated women. Hazard ratios were obtained from a Cox proportional hazards regression model. Time was included as an interaction term for the 2 periods in the unadjusted and adjusted analyses. Number of infants included: 21 643.

^b^ Adjusted for maternal age, maternal parity, maternal country of birth, maternal educational attainment as of 2019, and county of residence.

### Sensitivity Analyses

Results were robust in sensitivity analyses, although estimates were attenuated when restricting to infants born to Scandinavian-born women (eTables 1-6 in the Supplement). The results for the Omicron-dominated period when restricted to January 15 and later were similar to the main analyses (eTable 7 in the Supplement). Using the infant age as the time scale attenuated the association slightly (eTable 8 and eFigure 2 in the Supplement).

### Secondary Analyses

The proportion of infants hospitalized with COVID-19 as the main diagnosis before age 4 months or April 4, 2022, was 0.07% in both groups. The numbers were too low to perform formal comparative analyses by maternal vaccination status (Table 1).

In addition to the 21 463 infants in the main analysis, 2839 infants were born to women who only received 1 dose of an mRNA vaccine during the second or third trimester of pregnancy and at least 14 days before delivery. The timing of vaccine doses was differentially distributed by calendar time and time interval before birth between those who received only 1 vs a second or third dose in pregnancy (eFigures 3 and 4 in the Supplement), and the mean follow-up time was shorter. Among those born to women who received 1 dose of vaccine, 36 infants had a positive SARS-CoV-2 PCR test, including fewer than 5 during the Delta-dominated period. The aHR for a positive test in infants during the Omicron-dominated period born to women with 1 dose of vaccine compared with infants born to unvaccinated women was 0.72 (95% CI, 0.50-1.03) (eTable 9 in the Supplement).

Among 824 infants born to women who received their third vaccine dose during pregnancy, none had a positive SARS-CoV-2 test during the Delta-dominated period. The risk of a positive test was lower for the Omicron-dominated period for those with a third dose (aHR, 0.22; 95% CI, 0.12-0.43) compared with those with a second dose (aHR, 0.70; 95% CI, 0.59-0.83) (eTable 10 in the Supplement).

The proportion of infants who had at least 1 PCR test for SARS-CoV-2 during follow-up differed by maternal vaccination status: 2309 infants (19.4%) born to unvaccinated mothers and 1206 infants (12.4%) born to vaccinated mothers (Table 1). Corresponding incidence rates were 19.7 per 10 000 follow-up days among infants born to unvaccinated women and 15.1 per 10 000 follow-up days among infants born to vaccinated women.

## Discussion

This cohort study of all live births in Norway between September 1, 2021, and February 28, 2022, found that COVID-19 vaccination during pregnancy was associated with a reduced risk of an infant receiving a positive PCR test for SARS-CoV-2 during the first 4 months of life. This association was present during periods dominated by the Delta and Omicron variants, although it was stronger in the former. Results were robust in sensitivity analyses, although the number of cases during the Delta-dominated period was low in some of the subgroups. The association was somewhat attenuated when restricted to infants born to Scandinavian-born women.

It is not unexpected that maternal COVID-19 vaccination during pregnancy could reduce infant risk of COVID-19, as similar protective benefits against infant infection have been documented for pertussis and influenza vaccination during pregnancy in randomized clinical trials and observational studies.^2,3^ Because the newborn’s immune system is naive, with limited antibody response during the first months of life, an important protection against infection comes from maternally transferred antibodies.^2,3^ Infants are at higher risk of severe COVID-19 compared with older children.^1^ As to our knowledge no COVID-19 vaccines are licensed for use in children younger than 5 years, an added benefit of maternal vaccination during pregnancy could be a protection of infants against SARS-CoV-2 infection during the first months of life.^4^

We observed a lower risk of infection among infants born to women who received their third dose in pregnancy compared with the second, suggesting a stronger level of protection following the booster dose. This aligns with studies showing a waning of vaccine effect after the second dose unless a booster is received.^11^ Infants born to women with only 1 mRNA vaccine dose received during pregnancy also had a lower risk of a positive SARS-CoV-2 test than those born to unvaccinated women, but results were not statistically significant.

### Strengths and Limitations

The strengths of this study include the use of registry data covering the whole Norwegian population and many individuals vaccinated during pregnancy. Mandatory reporting to registries (including all COVID-19 vaccinations) limited potential selection bias and provided detailed information on clinical and sociodemographic variables. We believe our study results are generalizable to other pregnant populations. This assumption is strengthened by the fact that the findings align with the results from the US study examining maternal COVID-19 vaccination and risk of infant hospitalization for COVID-19.^6^

The limitations of this study included the lack of information on the infant’s test with a SARS-CoV-2 variant. However, there were distinct periods of dominance with the different variants in Norway during the study period, and we believe the defined periods capture risk with the different variants. The differences we observed in estimates for the Delta and Omicron-dominated periods support this, as the vaccines generally have been shown to be less effective against Omicron than Delta.^8,11^

Although we did not include vaccinations during the last 14 days of pregnancy or after pregnancy in the vaccinated group to allow for sufficient time of transplacental transfer of antibodies before birth,^22^ there could be a possible added effect of transfer of SARS-CoV-2 antibodies through breastmilk in these children,^23,24^ as more than 90% of infants in Norway are breastfed.^25,26^ We did not have individual-level information on breastfeeding and were unable to directly address whether this differed by maternal vaccination status.

We adjusted for potential confounders, which did not substantially affect the estimates. Still, there may be residual confounding because of healthy vaccinee bias^27^ or other unmeasured differences in characteristics between women who got vaccinated during pregnancy and those who did not.

The distribution of follow-up time in the vaccinated and unvaccinated groups varied across the 2 periods. We used calendar time as the underlying time scale in the analyses to ensure that comparisons were made on the same calendar days. This was important, as maternal vaccination status and risk of SARS-CoV-2 infection varied substantially over the study period. Although we did not have information on the number of household members or positive SARS-CoV-2 tests among them, we adjusted for maternal parity as a proxy.

We did not have information on disease symptoms in the infants. Thus, we could not assess the severity of the infections and whether this differed by maternal vaccination status. As the number of infants hospitalized for COVID-19 was low, we could not perform robust analyses to discern whether this differed by maternal vaccination status. We found that infants born to unvaccinated women were more likely to be tested for SARS-CoV-2, and this could be because of higher incidence of COVID-19 or a higher likelihood of symptomatic disease leading to testing. Although we cannot exclude differential test behavior according to maternal vaccination status, we believe it is unlikely. However, women who got vaccinated may have behaved differently (ie, taking more or fewer precautions to limit infant infection risk), which could have biased the estimates. Still, this is unlikely to account for all of the substantial reduction in risk that we observed.

## Conclusions

In this nationwide registry-based cohort study, we found that infants born to women who received a second or third COVID-19 vaccine dose during the last 2 trimesters of pregnancy had a lower incidence of SARS-CoV-2 infection within the first 4 months of life compared with infants born to unvaccinated women. The reduction in infant infection risk was greater during the Delta-dominated period compared with the Omicron-dominated period. The findings of this study provide early evidence to suggest that infants benefit from passive protection from SARS-CoV-2 infection following maternal COVID-19 vaccination during pregnancy.

## Notes

Supplement. **eTable 1. **Sensitivity analysis excluding infants born to women with Covid-19 prior to birth **eTable 2. **Sensitivity analysis of term-born infants **eTable 3. **Sensitivity analysis of infants born after a pregnancy conceived at least 42 gestational weeks prior to February 28, 2022 **eTable 4. **Sensitivity analysis of infants born to a Scandinavian-born woman **eTable 5. **Sensitivity analysis of infants born to first-time mothers **eTable 6. **Sensitivity analysis excluding infants born to women whose first vaccination dose was a non-mRNA vaccine **eTable 7. **Sensitivity analysis starting follow-up time on January 15, 2022 **eTable 8. **Sensitivity analysis using the age of the child as time-axis **eTable 9. **Sensitivity analysis including infants born to women who received only one mRNA vaccine dose during pregnancy **eTable 10. **Sensitivity analysis stratifying by second and third vaccine dose in pregnancy **eFigure 1. **Distributions by maternal vaccination status **eFigure 2. **Cumulative proportion of infants with a positive SARS-CoV-2 test by maternal vaccination and infant age **eFigure 3. **Calendar date of last vaccine dose in pregnancy **eFigure 4. **Time between birth and last vaccine dose in pregnancy
