# Supplementary material for: Association of COVID-19 Vaccination During Pregnancy With Incidence of SARS-CoV-2 Infection in Infants
Source: JAMA Intern Med. 2022 Jun 1;182(8):825–31. doi: 10.1001/jamainternmed.2022.2442 (PMC9161123; doi:10.1001/jamainternmed.2022.2442)
Supplement: Supplement. — eTable 1. Sensitivity analysis excluding infants born to women with Covid-19 prior to birth eTable 2. Sensitivity analysis of term-born infants eTable 3. Sensitivity analysis of infants born after a pregnancy conceived at least 42 gestational weeks prior to February 28, 2022 eTable 4. Sensitivity analysis of infants born to a Scandinavian-born woman eTable 5. Sensitivity analysis of infants born to first-time mothers eTable 6. Sensitivity analysis excluding infants born to women whose first vaccination dose was a non-mRNA vaccine eTable 7. Sensitivity analysis starting follow-up time on January 15, 2022 eTable 8. Sensitivity analysis using the age of the child as time-axis eTable 9. Sensitivity analysis including infants born to women who received only one mRNA vaccine dose during pregnancy eTable 10. Sensitivity analysis stratifying by second and third vaccine dose in pregnancy eFigure 1. Distributions by maternal vaccination status eFigure 2. Cumulative proportion of infants with a positive SARS-CoV-2 test by maternal vaccination and infant age eFigure 3. Calendar date of last vaccine dose in pregnancy eFigure 4. Time between birth and last vaccine dose in pregnancy [file jamainternmed-e222442-s001.pdf]

Supplemental Online Content

Carlsen EO, Magnus MC, Oakley L, et al. Association of COVID-19 vaccination during pregnancy with incidence of SARS-CoV-2 infection in infants. *JAMA Intern Med*. Published online June 1, 2022. doi:10.1001/jamainternmed.2022.2442

eTable 1. Sensitivity analysis excluding infants born to women with Covid-19 prior to birth

eTable 2. Sensitivity analysis of term-born infants

eTable 3. Sensitivity analysis of infants born after a pregnancy conceived at least 42 gestational weeks prior to February 28, 2022

eTable 4. Sensitivity analysis of infants born to a Scandinavian-born woman

eTable 5. Sensitivity analysis of infants born to first-time mothers

eTable 6. Sensitivity analysis excluding infants born to women whose first vaccination dose was a non-mRNA vaccine

eTable 7. Sensitivity analysis starting follow-up time on January 15, 2022

eTable 8. Sensitivity analysis using the age of the child as time-axis

eTable 9. Sensitivity analysis including infants born to women who received only one mRNA vaccine dose during pregnancy

eTable 10. Sensitivity analysis stratifying by second and third vaccine dose in pregnancy

eFigure 1. Distributions by maternal vaccination status

eFigure 2. Cumulative proportion of infants with a positive SARS-CoV-2 test by maternal vaccination and infant age

eFigure 3. Calendar date of last vaccine dose in pregnancy

eFigure 4. Time between birth and last vaccine dose in pregnancy

This supplemental material has been provided by the authors to give readers additional information about their work.

© 2022 Carlsen EO et al. *JAMA Internal Medicine.*

eTable 1: Sensitivity analysis excluding infants born to women with a positive SARS-CoV-2 PCR-test up to 14 days prior to giving birth. Number of infants included: 20,244. Time was included as an interaction term for the two time periods in the unadjusted and adjusted analyses.

	**Before January 1, 2022**						**After January 1, 2022**					
	**Live-born infants, No.**	**Follow-up time, days**	**Covid-19 positive test, No. (%)**	**Incidence rate, cases/10,000 days of follow-up**	**Unadjusted hazard ratio (95% CI)**	**Adjusted hazard ratio [a] (95% CI)**	**Live-born infants, No.**	**Follow-up time, days**	**Covid-19 positive test, No. (%)**	**Incidence rate, cases/10,000 days of follow-up**	**Unadjusted hazard ratio (95% CI)**	**Adjusted hazard ratio [a] (95% CI)**
**Unvaccinated**	9177	456298	144 (1.6)	3.2	Reference	Reference	5850	271506	314 (5.4)	11.6	Reference	Reference
**Vaccinated**	4669	206199	25 (0.5)	1.2	0.27 (0.18 to 0.41)	0.28 (0.18 to 0.43)	9253	526832	380 (4.1)	7.2	0.61 (0.52 to 0.71)	0.64 (0.54 to 0.76)

[a] Adjusted for maternal age, maternal parity, maternal country of birth, maternal educational attainment as of 2019, and county of residence.

© 2022 Carlsen EO et al. *JAMA Internal Medicine.*

eTable 2: Sensitivity analysis of term births only, excluding infants born before 37 gestational weeks. Number of infants included: 20,423. Time was included as an interaction term for the two time periods in the unadjusted and adjusted analyses.

	Before January 1, 2022						After January 1, 2022					
	**Live-born infants, No.**	**Follow-up time, days**	**Covid-19 positive test, No. (%)**	**Incidence rate, cases/10,000 days of follow-up**	**Unadjusted hazard ratio (95% CI)**	**Adjusted hazard ratio [a] (95% CI)**	**Live-born infants, No.**	**Follow-up time, days**	**Covid-19 positive test, No. (%)**	**Incidence rate, cases/10,000 days of follow-up**	**Unadjusted hazard ratio (95% CI)**	**Adjusted hazard ratio [a] (95% CI)**
**Unvaccinated**	9261	461794	139 (1.5)	3.0	Reference	Reference	6370	302516	338 (5.3)	11.2	Reference	Reference
**Vaccinated**	4414	194576	24 (0.5)	1.2	0.29 (0.19 to 0.45)	0.30 (0.19 to 0.47)	9026	514639	364 (4.0)	7.1	0.62 (0.53 to 0.72)	0.67 (0.56 to 0.79)

[a] Adjusted for maternal age, maternal parity, maternal country of birth, maternal educational attainment as of 2019, and county of residence.

© 2022 Carlsen EO et al. *JAMA Internal Medicine.*

eTable 3: Sensitivity analysis of infants conceived at least 42 weeks prior to end of inclusion to avoid oversampling of short pregnancies. Number of infants included: 19,495. Time was included as an interaction term for the two time periods in the unadjusted and adjusted analyses.

	**Before January 1, 2022**						**After January 1, 2022**					
	**Live-born infants, No.**	**Follow-up time, days**	**Covid-19 positive test, No. (%)**	**Incidence rate, cases/10,000 days of follow-up**	**Unadjusted hazard ratio (95% CI)**	**Adjusted hazard ratio [a] (95% CI)**	**Live-born infants, No.**	**Follow-up time, days**	**Covid-19 positive test, No. (%)**	**Incidence rate, cases/10,000 days of follow-up**	**Unadjusted hazard ratio (95% CI)**	**Adjusted hazard ratio [a] (95% CI)**
**Unvaccinated**	9743	485567	146 (1.5)	3.0	Reference	Reference	6178	296278	332 (5.4)	11.2	Reference	Reference
**Vaccinated**	4690	206715	25 (0.5)	1.2	0.29 (0.19 to 0.44)	0.30 (0.19 to 0.46)	8024	476946	360 (4.5)	7.5	0.65 (0.56 to 0.76)	0.70 (0.59 to 0.84)

[a] Adjusted for maternal age, maternal parity, maternal country of birth, maternal educational attainment as of 2019, and county of residence.

© 2022 Carlsen EO et al. *JAMA Internal Medicine.*

eTable 4: Sensitivity analysis of infants born to a Scandinavian-born woman. Number of infants included: 15,701. Time was included as an interaction term for the two time periods in the unadjusted and adjusted analyses.

	**Before January 1, 2022**						**After January 1, 2022**					
	**Live-born infants, No.**	**Follow-up time, days**	**Covid-19 positive test, No. (%)**	**Incidence rate, cases/10,000 days of follow-up**	**Unadjusted hazard ratio (95% CI)**	**Adjusted hazard ratio [a] (95% CI)**	**Live-born infants, No.**	**Follow-up time, days**	**Covid-19 positive test, No. (%)**	**Incidence rate, cases/10,000 days of follow-up**	**Unadjusted hazard ratio (95% CI)**	**Adjusted hazard ratio [a] (95% CI)**
**Unvaccinated**	6231	293624	56 (0.9)	1.9	Reference	Reference	3236	141039	163 (5.0)	11.6	Reference	Reference
**Vaccinated**	4066	180914	20 (0.5)	1.1	0.40 (0.24 to 0.67)	0.40 (0.24 to 0.68)	8333	473508	348 (4.2)	7.3	0.60 (0.50 to 0.73)	0.64 (0.52 to 0.78)

[a] Adjusted for maternal age, maternal parity, maternal country of birth, maternal educational attainment as of 2019, and county of residence.

© 2022 Carlsen EO et al. *JAMA Internal Medicine.*

eTable 5: Sensitivity analysis of infants born to a primiparous woman. Number of infants included: 9349. Time was included as an interaction term for the two time periods in the unadjusted and adjusted analyses.

	**Before January 1, 2022**						**After January 1, 2022**					
	**Live-born infants, No.**	**Follow-up time, days**	**Covid-19 positive test, No. (%)**	**Incidence rate, cases/10,000 days of follow-up**	**Unadjusted hazard ratio (95% CI)**	**Adjusted hazard ratio [a] (95% CI)**	**Live-born infants, No.**	**Follow-up time, days**	**Covid-19 positive test, No. (%)**	**Incidence rate, cases/10,000 days of follow-up**	**Unadjusted hazard ratio (95% CI)**	**Adjusted hazard ratio [a] (95% CI)**
**Unvaccinated**	4103	198332	37 (0.9)	1.9	Reference	Reference	2642	124631	111 (4.2)	8.9	Reference	Reference
**Vaccinated**	2177	93689	5 (0.2)	0.5	0.19 (0.08 to 0.49)	0.21 (0.08 to 0.55)	4387	248967	110 (2.5)	4.4	0.47 (0.36 to 0.62)	0.54 (0.40 to 0.73)

[a] Adjusted for maternal age, maternal parity, maternal country of birth, maternal educational attainment as of 2019, and county of residence.

© 2022 Carlsen EO et al. *JAMA Internal Medicine.*

eTable 6: Sensitivity analysis excluding infants born to vaccinated women who received the Oxford-AstraZeneca vaccine (ChAdOx1 nCoV-19) as their first vaccine dose.[a] Number of infants included: 21,083. Time was included as an interaction term for the two time periods in the unadjusted and adjusted analyses.

	**Before January 1, 2022**						**After January 1, 2022**					
	**Live-born infants, No.**	**Follow-up time, days**	**Covid-19 positive test, No. (%)**	**Incidence rate, cases/10,000 days of follow-up**	**Unadjusted hazard ratio (95% CI)**	**Adjusted hazard ratio [b] (95% CI)**	**Live-born infants, No.**	**Follow-up time, days**	**Covid-19 positive test, No. (%)**	**Incidence rate, cases/10,000 days of follow-up**	**Unadjusted hazard ratio (95% CI)**	**Adjusted hazard ratio [b] (95% CI)**
**Unvaccinated**	9759	485967	146 (1.5)	3.0	Reference	Reference	6728	320292	350 (5.2)	10.9	Reference	Reference
**Vaccinated**	4477	200158	24 (0.5)	1.2	0.28 (0.18 to 0.44)	0.29 (0.19 to 0.46)	9074	517800	372 (4.1)	7.2	0.64 (0.56 to 0.75)	0.68 (0.58 to 0.81)

[a] Exclusion of 380 infants whose mother's first vaccine dose was Oxford-AstraZeneca vaccine (ChAdOx1-S), but the second was an mRNA vaccine.

[b] Adjusted for maternal age, maternal parity, maternal country of birth, maternal educational attainment as of 2019, and county of residence.

© 2022 Carlsen EO et al. *JAMA Internal Medicine.*

eTable 7: Sensitivity analysis restricting follow-up time to start on January 15, 2022. Number of infants included: 15,230.

	After January 1, 2022					
	**Live-born infants, No.**	**Follow-up time, days**	**Covid-19 positive test, No. (%)**	**Incidence rate, cases/10,000 days of follow-up**	**Unadjusted hazard ratio (95% CI)**	**Adjusted hazard ratio [a] (95% CI)**
**Unvaccinated**	5808	253616	299 (5.1)	11.8	Reference	Reference
**Vaccinated**	9422	470863	354 (3.8)	7.5	0.65 (0.55 to 0.75)	0.59 (0.49 to 0.71)

[a] Adjusted for maternal age, maternal parity, maternal country of birth, maternal educational attainment as of 2019, and county of residence.

© 2022 Carlsen EO et al. *JAMA Internal Medicine.*

eTable 8: Sensitivity analysis using age of the infant as time axis. Number of infants included: 21,643. Time was included as an interaction term for the two time periods in the unadjusted and adjusted analyses.

	Before January 1, 2022						After January 1, 2022					
	**Live-born infants, No.**	**Follow-up time, days**	**Covid-19 positive test, No. (%)**	**Incidence rate, cases/10,000 days of follow-up**	**Unadjusted hazard ratio (95% CI) [a]**	**Adjusted hazard ratio [b] (95% CI)**	**Live-born infants, No.**	**Follow-up time, days**	**Covid-19 positive test, No. (%)**	**Incidence rate, cases/10,000 days of follow-up**	**Unadjusted hazard ratio (95% CI) [a]**	**Adjusted hazard ratio [b] (95% CI)**
**Unvaccinated**	9759	485967	146 (1.5)	3.0	Reference	Reference	6728	320292	350 (5.2)	10.9	Reference	Reference
**Vaccinated**	4696	206857	25 (0.5)	1.2	0.36 (0.24 to 0.56)	0.38 (0.24 to 0.58)	9616	546790	385 (4.0)	7.0	0.68 (0.59 to 0.79)	0.73 (0.62 to 0.87)

[a] Adjusted for week of birth.

[b] Adjusted for week of birth, maternal age, maternal parity, maternal country of birth, maternal educational attainment as of 2019, and county of residence.

© 2022 Carlsen EO et al. *JAMA Internal Medicine.*

eTable 9: Sensitivity analysis including infants born to vaccinated women who received only one mRNA vaccine dose during the second or third trimester of pregnancy. Number of infants included: 14,743. Time was included as an interaction term for the two time periods in the unadjusted and adjusted analyses.

	Before January 1, 2022						After January 1, 2022					
	Live-born infants, No.	Follow-up time, days	Covid-19 positive test, No. (%)	Incidence rate, cases/10,000 days of follow-up	Unadjusted hazard ratio (95% CI)	Adjusted hazard ratio [a] (95% CI)	Live-born infants, No.	Follow-up time, days	Covid-19 positive test, No. (%)	Incidence rate, cases/10,000 days of follow-up	Unadjusted hazard ratio (95% CI)	Adjusted hazard ratio [a] (95% CI)
**Unvaccinated**	9759	485967	146 (1.5)	3.0	Reference	Reference	6728	320292	350 (5.2)	10.9	Reference	Reference
**Vaccinated**	2215	80546	<5 (0.1)	0.2	0.10 (0.02 to 0.41)	0.11 (0.03 to 0.43)	1048	44919	34 (3.2)	7.6	0.68 (0.48 to 0.97)	0.72 (0.50 to 1.03)

[a] Adjusted for maternal age, maternal parity, maternal country of birth, maternal educational attainment as of 2019, and county of residence.

© 2022 Carlsen EO et al. *JAMA Internal Medicine.*

eTable 10: Sensitivity analysis stratifying by whether the mother received dose number 2 (n=8915) or 3 in pregnancy (n=824)[a]. Time was included as an interaction term for the two time periods in the unadjusted and adjusted analyses.

	Before January 1, 2022						After January 1, 2022					
	**Live-born infants, No.**	**Follow-up time, days**	**Covid-19 positive test, No. (%)**	**Incidence rate, cases/10,000 days of follow-up**	**Unadjusted hazard ratio (95% CI)**	**Adjusted hazard ratio [b] (95% CI)**	**Live-born infants, No.**	**Follow-up time, days**	**Covid-19 positive test, No. (%)**	**Incidence rate, cases/10,000 days of follow-up**	**Unadjusted hazard ratio (95% CI)**	**Adjusted hazard ratio [b] (95% CI)**
**Unvaccinated**	9759	485967	146 (1.5)	3.0	Reference	Reference	6728	320292	350 (5.2)	10.9	Reference	Reference
**Vaccinated with dose 2**	4666	206389	25 (0.5)	1.2	0.29 (0.19 to 0.44)	0.29 (0.19 to 0.45)	8792	501255	375 (4.3)	7.5	0.67 (0.57 to 0.77)	0.70 (0.59 to 0.83)
**Vaccinated with dose 3[a]**	30	468	0 (0)	0	N/A	N/A	824	45535	10 (1.2)	2.2	0.22 (0.11 to 0.41)	0.22 (0.12 to 0.43)

[a] Less than 5 women received the fourth dose in pregnancy and their infants are included in the 3-dose stratification.

[b] Adjusted for maternal age, maternal parity, maternal country of birth, maternal educational attainment as of 2019, and county of residence.

© 2022 Carlsen EO et al. *JAMA Internal Medicine.*

eFigure 1: Distributions according to maternal vaccination status (second/third dose during pregnancy versus none) during the whole time period.
A) Date of births, B) Date of positive SARS-CoV-2 PCR-test by 4 months of age, C) Age of child in days at the time of first positive SARS-CoV-2 test, D) Date of SARS-CoV-2 PCR-test, regardless of the result of that test.

A)

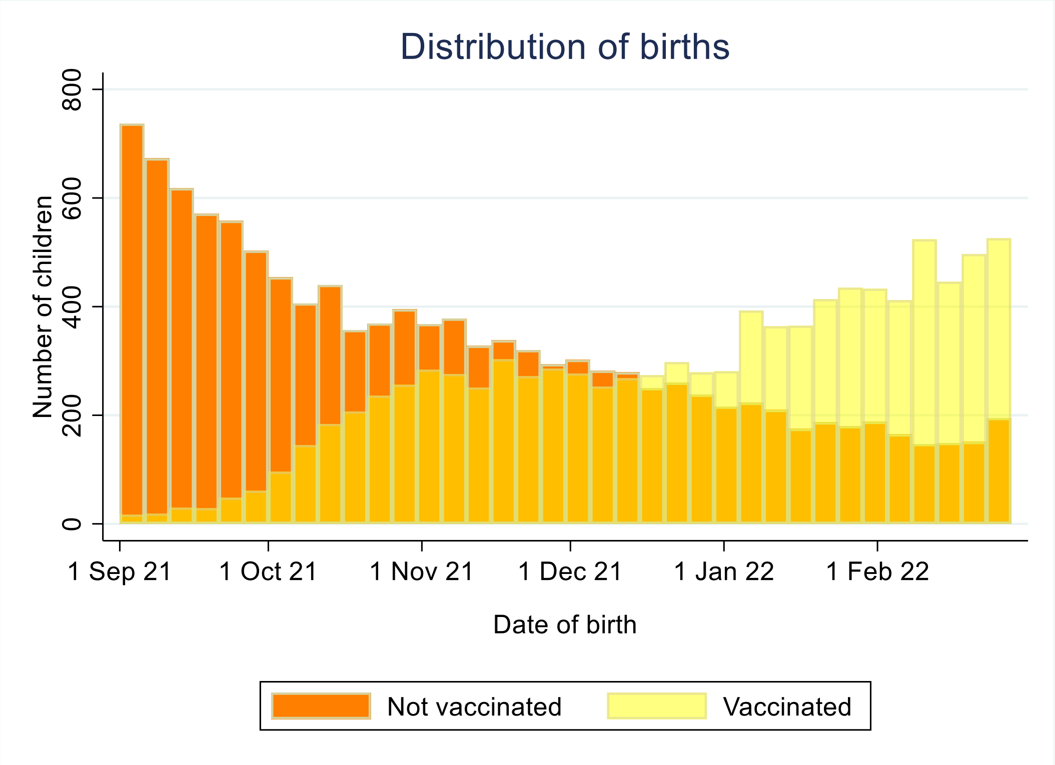

B)

© 2022 Carlsen EO et al. *JAMA Internal Medicine.*

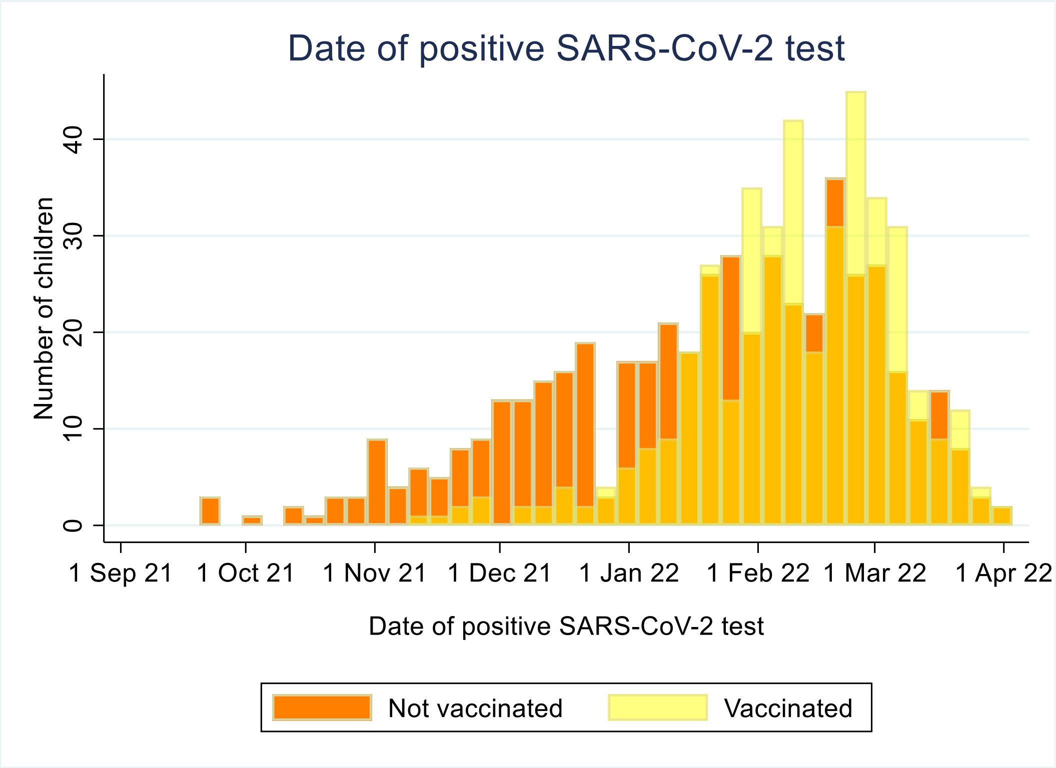

C)

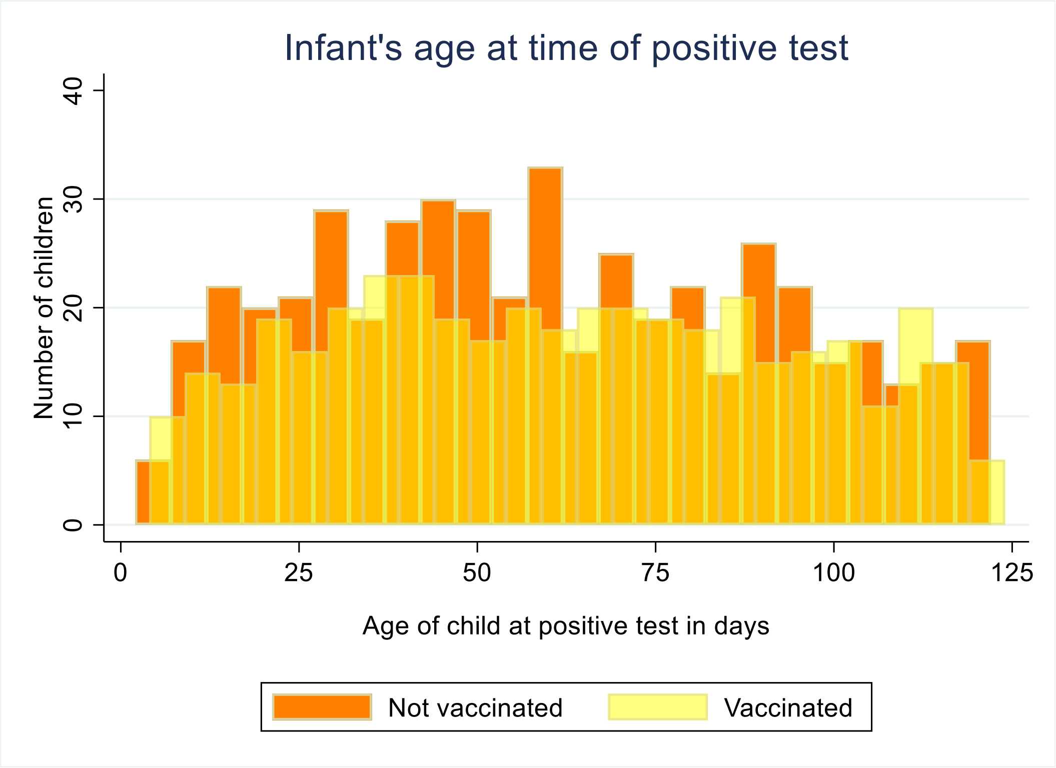

© 2022 Carlsen EO et al. *JAMA Internal Medicine.*

D)

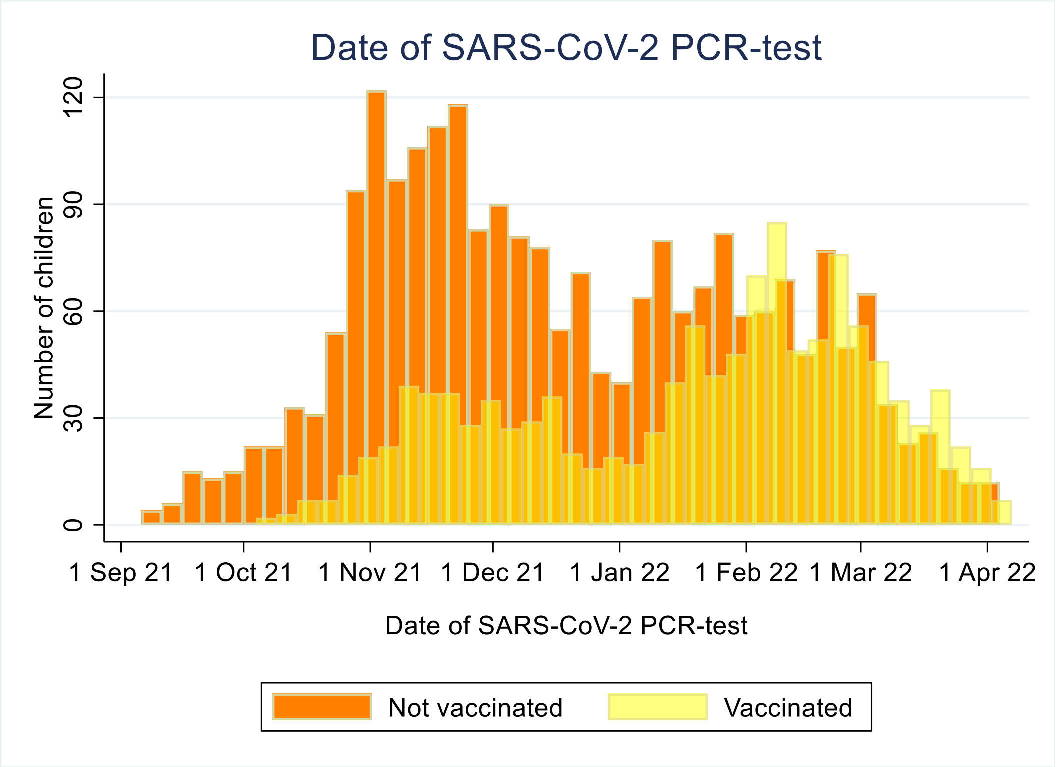

© 2022 Carlsen EO et al. *JAMA Internal Medicine.*

eFigure 2: Cumulative proportion of infants with a positive SARS-CoV-2 PCR-test by maternal vaccination status, using infant age as time axis

(A) Delta-dominated period: September 1 to December 31, 2021, and (B) Omicron-dominated period: January 1, to April 4, 2022.

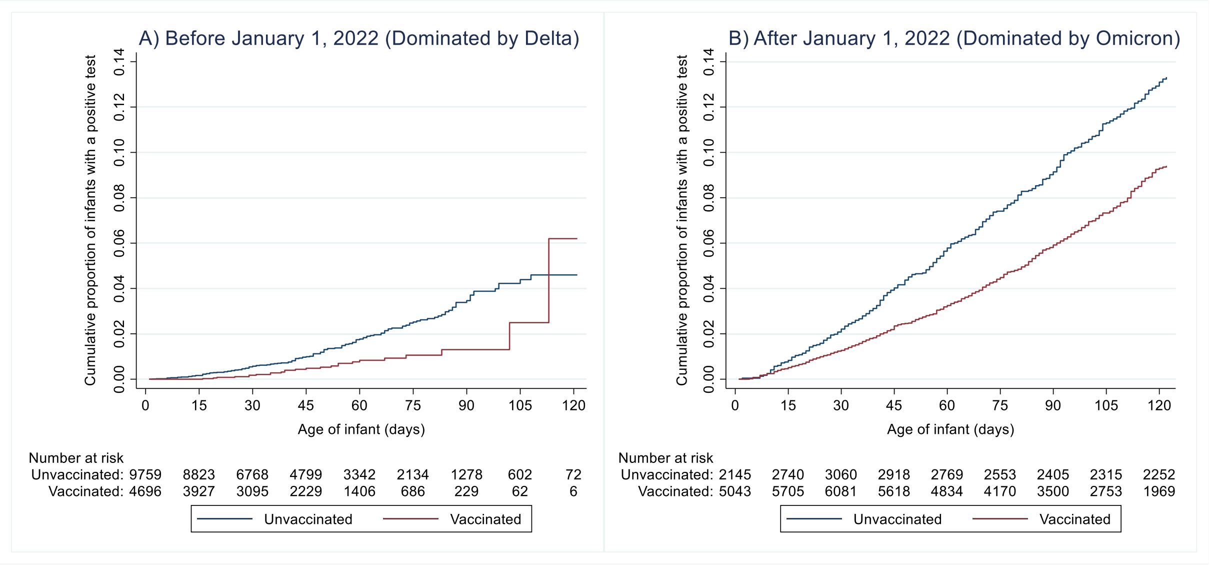

© 2022 Carlsen EO et al. *JAMA Internal Medicine.*

eFigure 3: Distribution of calendar time for date of last vaccine dose in pregnancy according to maternal vaccination status
A) Second or third dose in pregnancy, B) One vaccine dose during pregnancy

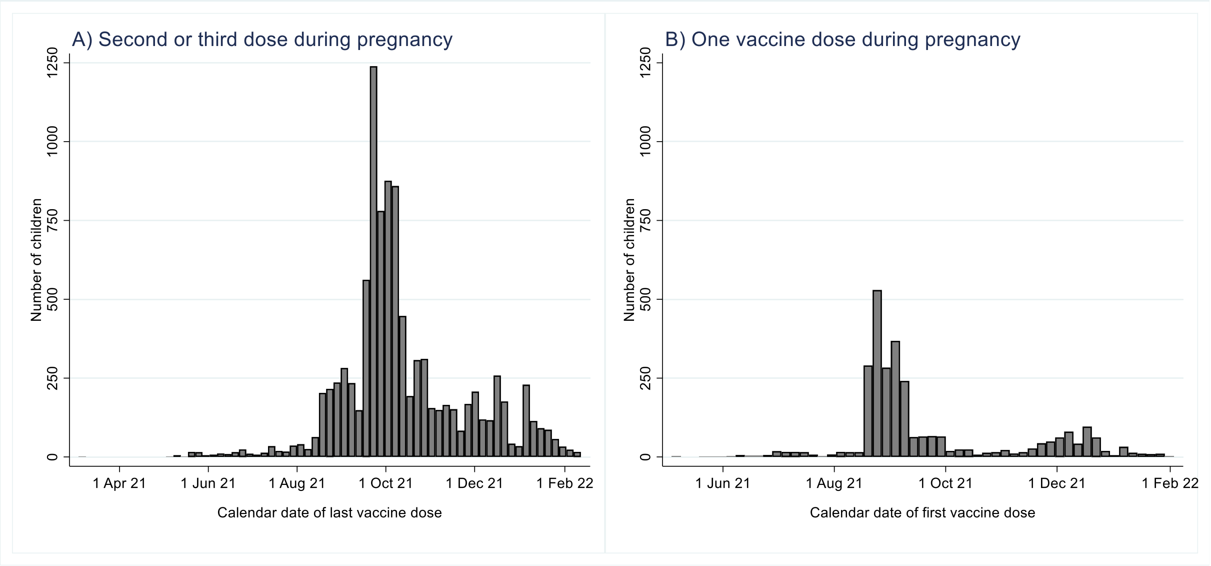

© 2022 Carlsen EO et al. *JAMA Internal Medicine.*

eFigure 4: Distribution of time between birth and last vaccine dose in pregnancy according to maternal vaccination status

A) Second or third dose in pregnancy, B) One vaccine dose during pregnancy

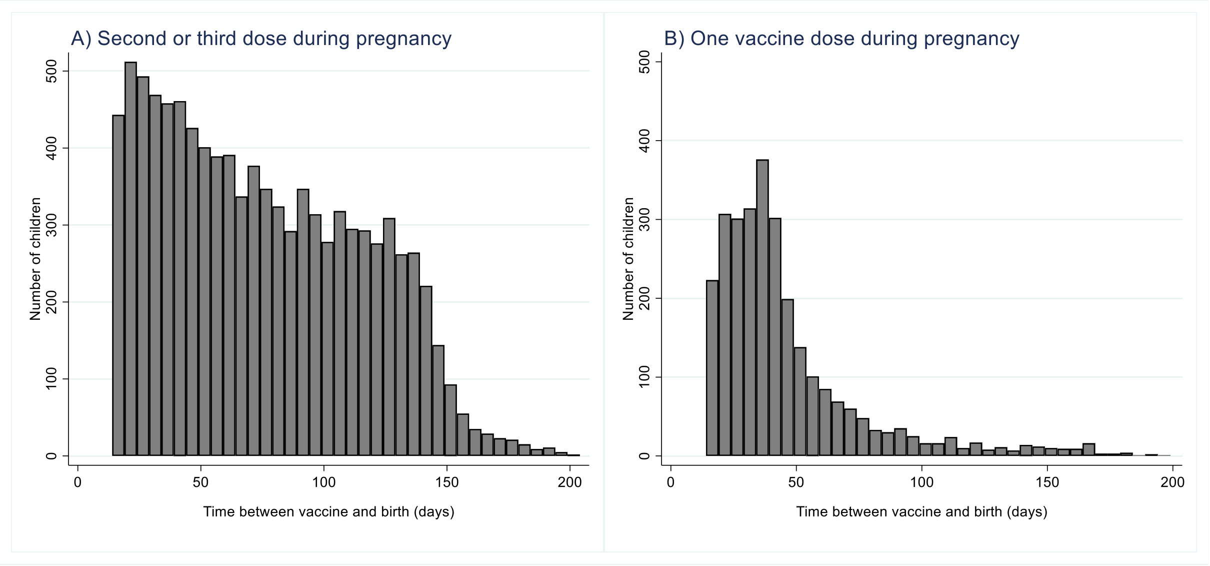

© 2022 Carlsen EO et al. *JAMA Internal Medicine.*
